# Effects of Fish Oil on HIV-Related Inflammation and Markers of Immunosenescence: A Randomized Clinical Trial

**DOI:** 10.1089/acm.2017.0222

**Published:** 2018-07-01

**Authors:** Barbara Swanson, Joyce Keithley, Linda Baum, Sue Leurgans, Oluwatoyin Adeyemi, Lisa L. Barnes, Mariana Mata, Anneliese Rosdil

**Affiliations:** ^1^ Department of Adult Health and Gerontological Nursing, Rush University College of Nursing, Chicago, IL.; ^2^ Department of Internal Medicine, Rush University Medical College, Chicago, IL.; ^3^ Department of Neurological Sciences, Rush University Medical College, Chicago, IL.; ^4^ Division of Infectious Diseases, John H. Stroger Hospital of Cook County, Chicago, IL.; ^5^ Department of Behavioral Sciences, Rush University Medical College, Chicago, IL.; ^6^ Rush Alzheimer's Disease Center, Rush University Medical Center, Chicago, IL.; ^7^ Children's Oncology Group, Los Angeles, CA.

**Keywords:** omega-3 fatty acids, HIV, immunosenescence

## Abstract

***Objective:*** To explore the safety and efficacy of fish oil to modulate parameters of inflammation and immunosenescence in HIV-infected older adults.

***Design:*** This study uses a randomized, controlled, double-blind clinical trial.

***Setting:*** The study was conducted in an outpatient HIV/AIDS clinic in a large urban Midwestern city in the United States.

***Subjects:*** A total of 37 clinically stable HIV-infected adults between the ages of 40 and 70 years of age participated.

***Interventions:*** Fish oil 1.6 g/day was administered for 12 weeks or placebo.

***Outcome measures:*** Inflammatory cytokine production, surface markers of immunosenescence, and adverse events were measured.

***Results:*** After 12 weeks of supplementation, there were no significant differences between the treatment and control groups on any measures of inflammation or immunosenescence in both CD4^+^ and CD8^+^ T lymphocytes. More participants in the treatment group reported adverse gastrointestinal events compared with the control group.

***Conclusions:*** A 12-week supplementation regimen of 1.6 g/day of fish oil did not favorably modulate parameters of inflammation or immune senescence in HIV-infected adults. Future studies should test agents that directly target mechanisms that underlie HIV-related inflammation to determine whether reducing inflammation can reverse immunosenescence.

## Introduction

Senescence of immune cells, characterized by diminished replicative capacity, has been observed in middle-aged persons treated with highly active antiretroviral therapy (HAART) who achieve immune reconstitution and undetectable viral loads.^1^ Despite the protection offered by HAART, the diminished capacity of these cells leads to the loss of anti-HIV cell-mediated immune responses, failure to normalize CD4^+^ T lymphocyte counts, and accelerated HIV disease progression.^2–4^

Senescent cells are characterized by the absence of the surface marker CD28, a receptor that is activated by costimulatory molecules on antigen-presenting cells to stimulate interleukin (IL)-2 production and clonal expansion.^5^ In states of chronic inflammation, T cells undergo continual proliferation and differentiation, leading to the formation of antigen-experienced cells that have a CD28^−^/CD57^−^ (intermediate senescence) or CD28^−^/CD57^+^ (terminal senescence) phenotype.^6^ These cells are characterized by replicative senescence due to shortened telomere lengths, and are also resistant to apoptosis, resulting in progressive accumulation of their numbers.^7,8^ HIV-related immunosenescence has been linked to persistent systemic inflammation that is postulated to be maintained by (1) the constant antigen burden imposed by HIV and other chronic viral copathogens, such as cytomegalovirus (CMV) and (2) HIV-induced disruption of intestinal epithelial integrity with subsequent translocation of gut microflora into the systemic circulation.^9,10^

Studies in murine models have shown that dietary supplements that modulate soluble mediators of inflammation can reverse age-associated declines in immune function. Supplementation of aged mice with one of two anti-inflammatory agents, α tocopherol (vitamin E) or dehydroepiandrosterone, before the administration of hemophilus influenza vaccine increased vaginal and fecal immunoglobulin A responses to levels seen in mature adult mice.^11^ Administration of the anti-inflammatory dietary supplement, *Cistanche deserticola*, reduced serum levels of IL-6 and extended the lifespan in a senescent-accelerated mouse model.^12^ These findings support the hypothesis that immunosenescence can be reversed by anti-inflammatory treatments.

Fish oil may be an effective treatment option for reducing HIV-related immunosenescence. Cold water fish are rich in the omega-3 highly unsaturated fatty acids (HUFA), eicosapentaenoic acid (EPA), and docosahexaenoic acid (DHA), which have anti-inflammatory effects.^13^ When consumed as fish or fish oil supplements, EPA and DHA replace arachidonic acid in cell membranes and inhibit the synthesis of proinflammatory arachidonic acid metabolites, such as prostaglandins and leukotrienes.^14^

A large body of literature has demonstrated that EPA and DHA reduce plasma concentrations of inflammatory cytokines.^15^ In clinical trials, fish oil supplementation has been associated with symptom relief and reductions in serum levels of proinflammatory cytokines in persons with rheumatoid arthritis and asthma,^16–18^ reduced plasma C-reactive protein (CRP) and IL-6 levels in persons with pancreatitis,^19^ and reduced flares in persons with Crohn's disease.^20^ Both the dose and duration of fish oil tested in randomized clinical trials (RCTs) have been highly variable. A recent meta-analysis of 68 RCTs of omega-3 fatty acid supplementation to favorably modulate inflammatory mediators in both healthy and chronic inflammatory disease populations (*N* = 4,601) found that total daily doses of EPA and DHA ranged from <1 to 5.4 g and duration ranged from 14 days to 1 year. Although omega-3 fatty acids were found to significantly lower concentrations of CRP, tumor necrosis factor-alpha (TNF-α), and IL-6, longer duration of supplementation was associated with greater effect size, especially when the duration exceeded 12 months.^21^

Although the Western diet is associated with lower omega-3 fatty acid intake than the Mediterranean diet, there are no federal intake recommendations for EPA or DHA for healthy adults. The American Heart Association recommends that healthy adults consume fatty fish at least two times per week and that persons with coronary heart disease consume 1 g/day of EPA and DHA combined as either fatty fish or supplements.^22^

Although fish oil decreases *in vitro* production of inflammatory cytokines in HIV-infected persons,^23,24^ to date, no studies have reported the effects of fish oil treatment on lymphocyte surface markers of immunosenescence in HIV-infected persons. A 12-week randomized controlled trial was conducted to test the effects of fish oil on intracellular inflammatory cytokine production and lymphocyte markers of immunosenescence in HAART-treated, clinically stable persons with HIV infection.

## Materials and Methods

### Design

The study used a randomized, controlled, double-blind clinical trial design and was registered in ClinicalTrials.gov (NCT02102724).

### Power analysis

The power analysis was based on a study of krill oil for reducing CRP levels in persons with arthritis (*N* = 90) that yielded an effect size (Cohen's *d*) of 1.2.^25^ A sample size of 37 was determined to yield a power of 0.80 to detect an effect size of 1.0 with a two-tailed α of 0.05.

### Setting and sample

The study was conducted between May 2014 and December 2015 at an American Midwest inner-city outpatient HIV/AIDS clinic located in a neighborhood that has been designated as medically underserved by the Health Resources Service Administration. Institutional review board approval was granted and the ethical standards of the Helsinki Declaration of 1975 were followed. All participants gave written informed consent before enrollment into the study.

Participants were eligible if they were HIV-infected adults between 40 and 70 years of age with a high sensitivity C-reactive protein (hsCRP) level of >2.0 mg/L, a CD4^+^ T lymphocyte count of at least 250 cells/mm^3^, a viral load of <75 copies/mL, and a history of stable antiretroviral therapy for at least 2 months. Participants were excluded from the study if they had a history of coronary artery disease or stroke; a platelet count <150,000 cells/mm^3^; a chronic condition known to affect lipid status (i.e., diabetes mellitus and familial hyperlipidemia); current use of fish oil supplements; use of medications or herbal therapies reported to influence bleeding parameters; documented active opportunistic infections or malignancies; serum creatinine >2.0 mg/dL or liver enzyme elevations greater than three times the upper limit of normal; low density lipoprotein cholesterol ≥120 mg/dL; or use of lipid-lowering medications.

### Procedures

Participants were recruited from the Research Core of the Rush Center of Excellence on Disparities in HIV and Aging (P20MD6886) using flyers and outreach efforts by the clinic's research coordinator. Participants completed five study visits. At the first two visits (2 weeks apart), medical history data and blood samples were collected to determine eligibility. At the third visit (2 weeks later), participants were randomized to the study arms and given oral and written instructions for the supplement. At the fourth visit (4 weeks after starting the supplement), safety parameters and adverse events were measured through blood samples and self-report. At the fifth visit (12 weeks after starting the supplement), blood samples and self-report data were collected to measure safety and efficacy.

### Intervention

Participants were randomized 1:1 to either the fish oil or placebo condition, using random numbers generated in S-PLUS, as adapted by Marsaglia et al.,^26^ in a random-length permuted block design.

Participants randomized to the fish oil condition received 12 weeks of 1.6 g/day of fish oil. Participants were instructed to take one gelcap twice daily with the morning and evening meal. Each gelcap contained 400 mg of EPA, 300 mg of DHA, and 100 mg of other omega-3 fatty acids (Carlson^®^ Fish Oil; Arlington Heights, IL). Similar doses and durations have been associated with improvements in cytokine profiles in HIV-infected persons^24^ and metabolic parameters in otherwise healthy persons with dyslipidemia.^27^ In addition, since data on the safety of fish oil for HIV-infected older adults were scant, a relatively low dose was tested. Participants randomized to the placebo condition received 1.0 g/day of oleic sunflower oil in gelcaps that were identical in appearance to the fish oil gelcaps. Participants were instructed to follow their usual dietary and activity patterns. Analyses of the fish oil and placebo gel caps confirmed both the composition and absence of rancidity and contaminants (ConsumerLab, Vernon, NJ). All participants and study personnel, except the statistician, were blinded to group assignment.

## Measures

### Demographics and lifestyle

Demographic and lifestyle variables, including age, gender, ethnicity, smoking, and HIV risk behaviors, were measured using a demographic questionnaire developed for a previous supplement study.^28^

### CD4^+^ T lymphocyte counts

CD4 T lymphocyte counts were quantified using a whole blood procedure for lysis of red blood cells and subsequent flow cytometric analysis of lymphocyte subsets.

### HIV RNA

Plasma HIV-1 RNA concentration was quantified using a polymerase chain reaction assay (COBAS^®^ Ampliprep/COBAS TaqMan^®^ HIV-1 Test; Roche Diagnostics, Mannheim, Germany) that had a lower level of detectability of 20 copies/mL of HIV-1.

### Liver function tests and serum creatinine

Liver function tests (LFTs) and serum creatinine were quantified using kinetic methods.

### hsCRP

hsCRP was measured using an immunochemiluminometric assay with a lower level of detectability of 0.1 mg/L.

### Intracellular cytokines

The percentage of stimulated CD4^+^ and CD8^+^ T lymphocytes that produced IL-6, TNF-α, and interferon-gamma (IFN-γ) was measured using flow cytometry. Peripheral blood mononuclear cells, obtained by density centrifugation, were washed and suspended at a concentration of 2 × 10^6^ cells/mL. Cells were added to a 24-well plate in a suspension that contained complete medium (RPMI medium +10% fetal bovine serum) with 1 μL/mL of brefeldin-A and 25 ng/mL of phorbol myristate acetate (PMA) and 500 ng/mL of ionomycin for IFN-γ analysis, or with 10 μg/mL of lipopolysaccharide (LPS) from *Escherichia coli* K-235 for TNF-α and IL-6 analysis. Unstimulated control wells contained complete medium with brefeldin-A only. The plate was incubated at 37°C for 4 h and then placed at 4°C overnight. The next morning, the cells were washed and resuspended in 1 mL of phosphate-buffered saline (PBS), and incubated for 30 min in the dark at room temperature (RT) with 1 μL/test of AQUA fixable viability dye (LifeTechnologies, Carlsbad, CA). Cells were washed and resuspended in fluorescence-activated cell sorting (FACS) buffer (0.2% bovine serum albumin +0.1% NaN_3_ in PBS) with 10 μL/test of Fc receptor block (Miltenyi Biotec, Inc., Auburn, CA) for 30 min at RT in the dark then washed in FACS buffer and incubated with PerCP Cy5.5 antihuman-CD8a and APC antihuman-CD4 (Tonbo Biosciences, San Diego, CA), PE Cy-7 antihuman CD28 and PB antihuman CD57 (BioLegend, San Diego, CA) for 30 min at 4°C in the dark. After this incubation, cells were washed in FACS buffer and fixed in 300 μL of 2% formaldehyde for 30 min at RT in the dark. Cell membranes were permeabilized by washing and incubating the cells with 500 μL of perm/wash buffer (BioLegend) for 30 min at RT in the dark. After this incubation, LPS-stimulated cells and their unstimulated controls were washed and resuspended in perm/wash buffer and incubated with PE antihuman IL-6 and fluorescein isothiocyanate antihuman TNF-α for 30 min at RT in the dark. Similarly, PMA/I-stimulated cells and their unstimulated controls were incubated with PE antihuman IFN-γ. Appropriate isotype controls were used for each intracellular cytokine antibody (all from Biolegend). Cells were washed again and resuspended in FACS buffer and analyzed using an LSRFortessa (BD Biosciences, San Jose, CA) flow cytometer. Dead cells were excluded from analysis by gating on AQUA viability dye negative cells.

### Immunosenescence parameters

Immunosenescence was measured at baseline and week 12 by quantifying the percentage of CD4 and CD8 cells that were CD28^+^/CD57^+^, CD28^−^/CD57^−^, and CD28^−^/CD57^+^ using the stimulation and staining protocol already explained. Analyses were performed after stringent gating on singlet live (AQUA^−^) CD4^+^ or CD8^+^ T cells. CD28 and CD57 expression levels were evaluated by using positive gates on live CD4^+^ and CD8^+^ cells. All gating analysis was performed using FlowJo Software (TreeStar, Inc., Ashland, OR).

### Adherence

Adherence to the fish oil condition was measured by quantifying the percentage of omega-3 HUFA in the total plasma HUFA pool using gas chromatography.^29^ The assay was performed by Lipid Technologies (Austin, MN) using dried blood spots.

### Data analysis

All analyses were performed on an intent-to-treat basis using SAS (Version 9.3). Measures of central tendency were calculated for all baseline demographic and clinical variables and group differences were assessed using Wilcoxon rank sum tests for continuous variables and chi-square for categorical variables. To determine group differences in outcomes for the 12-week supplementation period, the change scores of the two groups were compared using Wilcoxon rank sum tests. The number of self-reported adverse events was summed and group differences were tested using chi-square analyses. Statistical significance was set at *p* < 0.05.

## Results

**FIG. 1.**
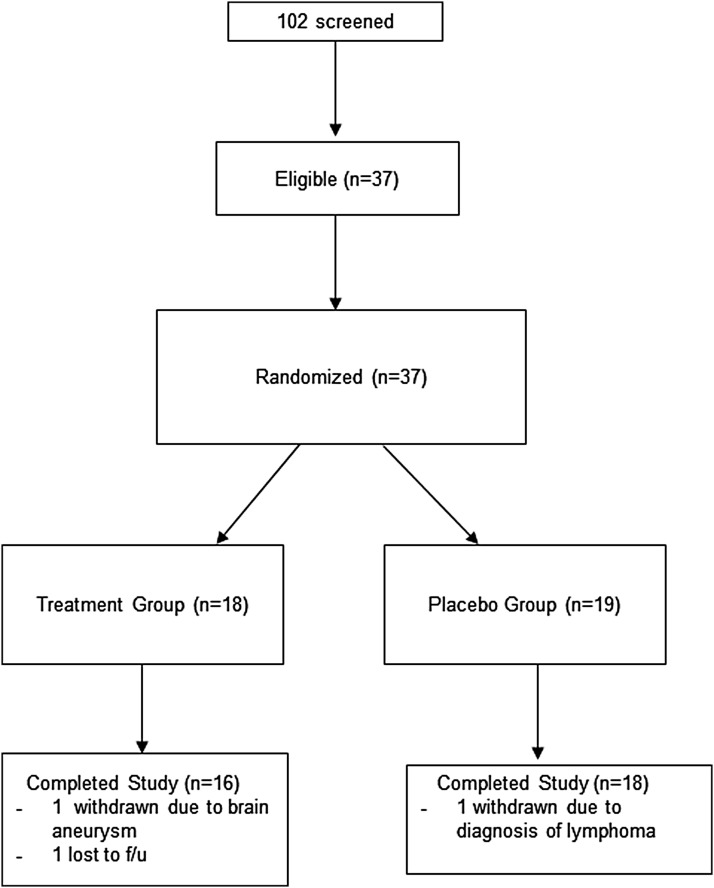
Study design and flow diagram.

A total of 102 participants were screened for eligibility and 37 were randomized and entered into the analyses (Fig. 1). Most of the ineligible participants did not meet the study's hsCRP inclusion criterion (>2.0 mg/L). Baseline demographic and clinical data for the randomized participants are shown in Table 1. The groups did not significantly differ on any baseline demographic or clinical measures (all *p*-values >0.05) except smoking, which was more frequent among participants in the placebo group. In addition, the groups did not differ at baseline with respect to cytokine production or senescent phenotype.

**Table 1.** Demographic and Clinical Characteristics of the Sample (*N* = 37)

	*Fish oil*	*Placebo*	
	*(*n* = 18)*	*(*n* = 19)*	p-Value^*^
Age (years) [median (*M*, SD)]	52 (54.5, 5.9)	55.5 (53, 6.1)	0.33
Gender (*n*; %)			0.87
Male	9 (50%)	10 (53%)	
Female	9 (50%)	9 (47%)	
Ethnicity (*n*; %)			0.29
African American	15 (83%)	18 (95%)	
Caucasian	3 (11%)	1 (5%)	
HIV risk behaviors (*n*; %)^a^			
Heterosexual contact	10 (55.5%)	14 (74%)	0.25
Same sex contact	5 (27.8%)	4 (21%)	0.63
Injection drug use	3 (16.7%)	2 (10.5%)	0.56
Blood transfusion	0	1 (5.3%)	0.32
Current smoker (*n*; %)	9 (50%)	15 (79%)	0.02^*^
CD4^+^ T lymphocyte count (cells/mm^3^) [median (*M*, SD)	522 (797, 602)	521 (672, 363)	0.68
HIV RNA (copies/mL) [median (*M*, SD)]	0	0	NA
Liver function (IU/L) [median (*M*, SD)]			
ALT	15 (18.4, 9.8)	22.5 (24.6, 12.0)	0.13
AST	22.2 (20, 8.3)	25 (27.2, 9.4)	0.08
Creatinine (mg/dL) [median (*M*, SD)]	0.94 (1.0, 0.27)	0.92 (0.94, 0.17)	0.67
hsCRP (mg/L) [median (*M*, SD)] [median (*M*, SD)]	7.36 (3.32, 12.1)	6.65, 6.23 (3.9)	0.16
EPA (% plasma concentration) [median (*M*, SD)]	0.42 (0.42, 0.12)	0.50 (0.49, 0.16)	0.17
DHA (% plasma concentration) [median (*M*, SD)]	2.03 (2.23, 0.72)	2.05 (2.01, 0.51)	0.57
Omega-3 HUFA (%) [median (*M*, SD)]	25 (19, 4.7)	18 (18.1, 2.1)	0.92

^a^ Total frequency >100% as participants reported more than one risk behavior.

^*^ *p*-Values from Wilcoxon rank sum tests for continuous variables and chi-square tests for categorical variables.

ALT, alanine aminotransferase; AST, aspartase aminotransferase; EPA, eicosapentaenoic acid; DHA, docosahexaenoic acid; HUFA, highly unsaturated fatty acids; hsCRP, high sensitivity C-reactive protein; NA, not applicable; SD, standard deviation.

At week 12, the treatment group showed significantly higher percentages of EPA, DHA, and omega-3 HUFA in the total plasma HUFA pool, suggesting that the treatment group was adherent to the fish oil regimen (Table 2); however, five participants in the fish oil group did not show an increase in plasma omega-3 HUFA at week 12. There were no statistically significant differences between the groups in change scores from baseline to week 12 for the safety measures (CD4^+^ T lymphocyte counts, HIV RNA, LFTs, and creatinine). Eight participants in the fish oil group reported adverse gastrointestinal events (i.e., nausea and diarrhea) compared with four participants in the placebo group (*χ*^2^ = 185; *p* < 0.0005).

**Table 2.** Change Scores in Plasma Fatty Acids From Baseline to Postsupplementation (*N* = 37)

	*Fish oil (*n* = 18)*				*Placebo (*n* = 19)*			
	*MedianΔ*	*MeanΔ*	*IQR*	*95% CI*	*MedianΔ*	*MeanΔ*	*IQR*	*95% CI*
*Fatty acids (%)*								
Omega-3 HUFA^*^	7.0	6.2	−1.0–11.0	2.4–10.1	0	−0.65	−2.0–0	−1.7–0.36
EPA^**^	0.81	3.18	0.06–1	−1.97–8.3	0.08	0.06	−0.16–0.2	−0.04–0.17
DHA^***^	0.43	0.67	0.05–1.45	0.15–1.2	−0.12	−0.13	−0.27–0.07	−0.27–0.01

*p*-Values for median score change: ^*^p = 0.008, ^**^p = 0.02, ^***^p = 0.002.

CI, confidence interval; DHA, docosahexaenoic acid; EPA, eicosapentaenoic acid; HUFA, highly unsaturated fatty acids; IQR, interquartile range.

There were no differences in change scores between baseline and week 12 in the percentage of stimulated CD4^+^ or CD8^+^ T lymphocytes that produced IL-6, TNF-α, or IFN-γ (all *p*-values >0.05). Similarly, there were no changes in the percentage of CD4^+^ or CD8^+^ T lymphocytes that expressed senescent phenotypes (Tables 3 and 4).

**Table 3.** Baseline and Postsupplementation CD4^+^ and CD8^+^ Lymphocytes: Cytokine Production and Senescence Markers (*N* = 37)

	*Fish oil (*n* = 18)*		*Placebo (*n* = 19)*	
	*Baseline*	*After 12 weeks*	*Baseline*	*After 12 weeks*
CD4^+^ cells (% positive)^a^ [median (*M*, SD)]				
Cytokines				
IL-6	0.20 (0.45, 0.55)	0.21 (0.92, 1.8)	0.19 (0.33, 0.50)	0.31 (0.52, 0.50)
IFN-γ	2.89 (11.5, 18.4)	8.50 (13.9, 17.3)	9.65 (12.4, 12.8)	12.23 (16.4, 19.6)
TNF-α	0.07 (0.10, 0.12)	0.48 (0.11, 0.24)	0.08 (0.13, 0.15)	0.05 (0.08, 0.08)
Senescence markers				
CD28^+^/CD57^−^	93.2 (91.0, 7.1)	94.0 (92.6, 6.7)	90.2 (85.4, 17.9)	92.1 (89.3, 7.4)
CD28^−^/CD57^−^	4.40 (5.5, 6.3)	1.99 (3.0, 2.8)	4.43 (5.2, 2.9)	3.57 (3.8, 2.4)
CD28^−^/CD57^+^	0.54 (1.4, 1.7)	0.20 (2.2, 4.6)	1.52 (6.12, 17.4)	1.88 (4.0, 5.5)
CD8^+^ cells (% positive)^a^ [median (*M*, SD)]				
Cytokines				
IL-6	0.07 (0.21, 0.30)	0.04 (0.14, 0.22)	0.07 (0.12, 0.14)	0.06 (0.17, 0.43)
IFN-γ	22.1 (32.0, 23.4)	29.8 (35.0, 23.9)	25.9 (30.7, 19.1)	40.2 (35.9, 24.3)
TNF-α	0.45 (0.09, 0.14)	0.02 (0.04, 0.06)	0.01 (0.50, 0.07)	0.03 (0.05, 0.07)
Senescence markers				
CD28^+^/CD57^−^	53.85 (51.1, 13.9)	50.2 (50.5, 16.5)	64.1 (55.9, 19.5)	50.0 (51.0, 18.9)
CD28^−^/CD57^−^	16.1 (16.8, 7.9)	14.0 (13.6, 8.1)	14.4 (13.6, 7.2)	13.8 (14.8, 2.5)
CD28^−^/CD57^+^	15.1 (22.2, 12.8)	21.0 (19.5, 13.1)	13.6 (17.3, 12.1)	14.4 (19.0, 14.6)

^a^ All *p*-values for Wilcoxon rank sum tests for baseline intergroup differences and intergroup change scores were >0.05.

IFN-γ, interferon-gamma; IL, interleukin; SD, standard deviation; TNF-α, tumor necrosis factor-alpha.

**Table 4.** Change Scores From Baseline to Postsupplementation in Cytokine Production and Senescence Markers (*N* = 37)

	*Fish oil (*n* = 18)*				*Placebo (*n* = 19)*			
	*MedianΔ*	*MeanΔ*	*IQR*	*95% CI*	*MedianΔ*	*MeanΔ*	*IQR*	*95% CI*
CD4^+^ cells (% positive)								
Cytokines								
IL-6	−0.003	0.43	−0.37–0.27	−1.5–0.63	0.15	0.29	−0.42–0.09	−0.58–0.01
IFN-γ	0.66	5.02	−16.7–6.64	−16.8–6.8	0.58	3.76	−12.1–7.8	−15.1–7.5
TNF-α	−0.01	0.01	−0.03–0.08	−0.16–0.14	−0.001	−0.05	−0.019–0.18	−0.03–0.14
Senescence markers								
CD28^+^/CD57^−^	0.70	1.87	−3.90–1.60	−2.8–6.5	1.45	4.22	−4.00–0.20	−3.5–11.9
CD28^−^/CD57^−^	−1.40	−2.83	−1.16–4.14	−6.9–1.2	−2.23	−1.56	0.18–3.98	−3.0 to −0.08
CD28^−^/CD57^+^	−0.19	0.69	−0.36–0.75	−1.2–2.5	−0.14	−2.41	−0.38–0.96	−9.6–4.8
CD8^+^ cells (% positive)								
Cytokines								
IL-6	−0.04	−0.10	−0.02–0.09	−0.01–0.21	0.001	0.08	−0.08–0.08	−0.31–0.16
IFN-γ	0.75	5.55	−38.1–23.9	−24.1–13.0	7.60	5.59	−15.7–3.50	−19.0–7.85
TNF-α	−0.02	−0.06	0.001–0.05	−0.001–0.05	0.01	0.004	−0.04–0.04	−0.04–0.03
Senescence markers								
CD28^+^/CD57^−^	1.30	−1.58	−11.8–15.3	−10.8–7.7	−0.15	−4.63	−13.0–20.1	−19.6–10.4
CD28^−^/CD57^−^	−3.73	−3.60	−1.65–7.04	−7.7–0.5	−0.42	0.79	−4.2–9.4	−4.8–6.4
CD28^−^/CD57^+^	−4.95	−2.37	−3.12–7.19	−8.7–4.0	−2.12	1.96	−12.32–7.0	−6.2–10.1

CI, confidence interval; IFN-γ, interferon-gamma; IL, interleukin; IQR, interquartile range; TNF-α, tumor necrosis factor-alpha.

To determine whether the nonadherent participants (*n* = 5) in the fish oil group affected the findings, the cytokine and senescent data were reanalyzed excluding those five participants. No significant differences were found between the two groups' change scores for any cytokine or immunosenescence variables (all *p*-values >0.05).

## Discussion

To the authors' knowledge, this study is the first to test the effects of fish oil to favorably modulate parameters of inflammation and immunosenescence in HIV-infected persons. No differences were found between the groups in the expected direction for any outcome measures, suggesting a lack of beneficial effect of fish oil at the tested dose and duration to modulate markers of inflammation and immunosenescence.

It is unlikely that nonadherence to the supplement accounted for the nonsignificant findings given the differences in the two groups' plasma individual and total omega-3 HUFA percentages at 12 weeks. It is possible that the fish oil dose and/or treatment duration were inadequate to reduce inflammatory cytokines in persons with HIV infection, which is characterized by persistently high levels of inflammatory cytokines even in HAART-treated persons who achieve durable viral suppression.^7^ Previous clinical trials of omega-3 fatty acids in persons with chronic inflammatory conditions, such as asthma, systemic lupus erythematosus, and rheumatoid arthritis, have found clinical improvement and anti-inflammatory effects with doses >3 g/day.^18,30,31^ A lower dose (1.6 g/day) was tested and found that a substantial number of participants in the treatment group reported adverse gastrointestinal events (8 of 18). Thus, higher doses might not be indicated in this population, since gastrointestinal events can potentially reduce adherence to antiretroviral medications and lead to loss of viral suppression.

It is unclear whether the high number of cigarette smokers in the sample affected the findings. Smoking has differential effects on lipid metabolism. Cigarette smoke contains high levels of oxidants and has been shown to induce peroxidation of plasma lipids, an indicator of oxidative stress.^32^ Conversely, cigarette smoke is associated with increased transport of polyunsaturated fatty acids (PUFA) into the plasma, which may offset the peroxidative PUFA loss.^33^ Additional studies are needed to clarify the influence of cigarette smoke on the anti-inflammatory effects of PUFA, such as omega-3 fatty acids.

The authors also failed to find an effect for fish oil for reducing the percentage of T lymphocytes that expressed senescent phenotypes. Fish oil may not directly affect the mechanisms that underlie immunosenescence, such as persistent infection with chronic copathogens such as CMV, which has been associated with immunosenescence and mortality in healthy adults.^34,35^ In a placebo-controlled trial of HIV-infected persons with asymptomatic CMV infection, the antiviral drug valganciclovir reduced both hsCRP levels and number of activated CD8^+^ T lymphocytes,^36^ which, in turn, have been associated with CD57 expression.^37^ Other agents that directly target immunosenescence mechanisms should be tested in future studies. Candidate agents include growth hormone, which has been shown to increase thymopoiesis and the number of naive CD4^+^ T lymphocytes in HIV-infected persons,^38^ and the probiotic *Lactobacillus*, which is known to reduce intestinal inflammation and maintain gut barrier integrity.^39^

The sample was a major strength of this study. A well-characterized sample of clinically stable HIV-infected older adults was recruited and enrolled who showed evidence of systemic inflammation despite durable viral suppression. Participants were largely medically underserved and socially/economically disadvantaged minorities who have been historically underrepresented in clinical trials and are at high risk for health outcome disparities. Other strengths include the objective measurement of adherence using plasma HUFA concentrations and the compositional analyses of the fish oil and placebo.

This study was limited by the relatively small sample size that precluded testing multiple fish oil doses; the single dose tested may not have been high enough to modulate inflammation or lymphocyte senescent markers. In addition, a *post hoc* analysis using change in the percentage of stimulated CD4^+^ T lymphocytes that produced intracellular TNF-α as the outcome showed that this sample size (*N* = 37) had a power of 0.14 to detect an effect size of 0.3. Thus, this study was underpowered to detect smaller effect sizes. A second limitation was the supplementation duration that precluded detecting effects that may occur after 12 weeks and limited conclusions about long-term safety.

## Conclusions

The findings suggest that fish oil supplementation for clinically stable HIV-infected older adults is not associated with adverse immunological or virological events, but is associated with mild gastrointestinal events. At the dose and duration tested, fish oil supplementation did not favorably modulate parameters of inflammation or immunosenescence. Future studies should test agents that directly target mechanisms underlying HIV-related inflammation to determine whether reducing inflammation can reverse immunosenescence.
